# *Astragalus membranaceus*, *Nigella sativa,* and *Perilla frutescens* as Immunomodulators—Molecular Mechanisms and Clinical Effectiveness in Allergic Diseases

**DOI:** 10.3390/cimb46080533

**Published:** 2024-08-17

**Authors:** Maja Bival Štefan

**Affiliations:** Department of Pharmacognosy, Faculty of Pharmacy and Biochemistry, University of Zagreb, 10000 Zagreb, Croatia; maja.bival@pharma.unizg.hr

**Keywords:** herbal immunomodulators, allergy, rhinitis, asthma, *Astragalus membranaceus*, *Nigella sativa*, *Perilla frutescens*

## Abstract

Plants are the source of numerous remedies in modern medicine, and some of them have been studied due to their potential immunomodulatory activity. *Astragalus membranaceus* Fisch. ex Bunge (*A. membranaceus*), *Nigella sativa* L. (*N. sativa*), and *Perilla frutescens* (L.) Britton (*P. frutescens*) are plant species used in traditional medicine for the treatment of various diseases. Their potential to act as immunomodulatory, anti-inflammatory, and anti-allergic agents makes them interesting for investigating their clinical potential in alleviating the symptoms of allergic diseases. Allergy affects a large number of people; according to some sources more than 30% of the world population suffer from some type of allergic reaction, with pollen allergy as the most common type. Treatment is usually pharmacological and may not be completely effective or have side effects. Thus, we are seeking traditional medicine, mostly medicinal plants, with promising potential for alleviating allergy symptoms. A literature overview was conducted employing databases such as Scopus, PubMed, Web of Science, Springer, and Google Scholar. This manuscript summarizes recent in vivo preclinical and clinical studies on three species with immunomodulatory activity, provides a comparison of their anti-allergic effects, and underlines the potential of their application in clinical practice. The obtained results confirmed their efficacy in the in vivo and clinical studies, but also emphasize the problem of phytochemical characterization of the species and difference between tested doses. More clinical trials with standardized protocols (defined active molecules, dosage, side effects) are required to obtain safe and effective herbal drugs.

## 1. Introduction

Hypersensitivity reactions are divided into four distinct groups based on their mechanism. Type I (immediate hypersensitivity) is mediated by allergen-specific immunoglobulin E (IgE), which is associated with the high-affinity receptors of basophils and mast cells. Allergic rhinitis, conjunctivitis, and allergic asthma are caused by type I hypersensitivity reactions. Type II reactions are cytotoxic reactions characterized by IgG and IgM antibodies against cell surface antigens found on circulating blood cells or epithelial cells. This process leads to phagocytosis and cytotoxicity. Type III reactions compromise formation of immune complexes by binding of IgG and IgM antibodies to antigens. These reactions can damage the organs due to deposition of complexes in the tissue. Delayed reactions (type IV) involve T cells as major effector cells, which cause damage directly or through the production or release of reactive oxygen species, lysosomal enzymes, and inflammatory cytokines [1,2]. 

Allergy can be defined as an undesirable overreaction of the immune system to normally harmless compounds. Due to the incapability of the immune system to distinguish harmless from pathological substances, it reacts on the harmless substances resulting in symptoms like sneezing, swelling of the mucous membrane, and finally asthma. In some cases, it can lead to life-threatening conditions [3].

The hypersensitive allergy reactions we recognize as allergic diseases of individual organs including nose (allergic rhinitis), sinuses (allergic sinusitis), eyes (allergic conjunctivitis), lower airways (allergic asthma), and skin (atopic dermatitis or eczema) [4,5,6].

Allergy affects a large number of people; according to some sources more than 30% of the world population suffer from some type of allergic reaction with increasing prevalence worldwide. The prevalence of allergic diseases is high in developed countries, but an increase in allergic diseases incidence has been recorded in developing countries, which could be related to a shift in lifestyle towards Western customs [7]. Allergic reactions include reactions to different substances from our environment, mainly house dust mites, animal dander, food allergens and, as the most common one, pollen [8].

Allergic rhinitis, associated with asthma, as well as conjunctivitis, are the most widespread inflammatory diseases affecting more than 500 million people worldwide, which makes it a global health problem [7].

Civilizational development and the Western way of life brought changes in hygiene habits, resulting in reduced exposure to parasites, bacterial, and viral substances, which is possibly part of the answer to why in the earliest age of the child, there is a slow development of the balance between Th1 and Th2 responses of lymphocytes. The “hygiene hypothesis” was first introduced in the 1970s by an observation that atopy and parasitic infections share elevated IgE, but never occur at the same time. Consequently, the lack of infections was associated with disturbed immunity. Th1/Th2 paradigm explains the inverse relationship of viral infections and atopy. Th1 cells produce IL-2 and INF-γ, responsible for antiviral reaction, while Th2 cells secrete IL-4 and IL-5 which induce IgE and eosinophilia. If there are no viral infections, Th2 polarization occurs. The mast cell saturation theory implicates that upon parasitic infection, the IgE receptors on the surface of mast cells are saturated and thereby disable binding of allergen-specific IgE, resulting in the prevention of an allergic reaction. In general, the hygiene hypothesis emphasizes that our immune system was adapted to ubiquitous microorganisms, and excessive hygiene resulted in an imbalanced immunoregulation causing various immune disorders, such as allergy [5]. 

In the modern way of life, we have reduced exposure to parasites, and are facing environmental pollution contributing to allergens becoming more aggressive and penetrating more easily through the mucous membrane of the respiratory system [9,10]. We are spending more time in the artificially ventilated indoor environment, thus being more exposed to dust mites, mold, cigarettes smoke, and humidity. A fast lifestyle and exposure to stress inevitably contribute to changes in the body’s immune response. A specific person’s immune response can be affected by the duration of exposure to the allergens and pollution as well as living conditions [11,12,13]. 

**Figure 1:**
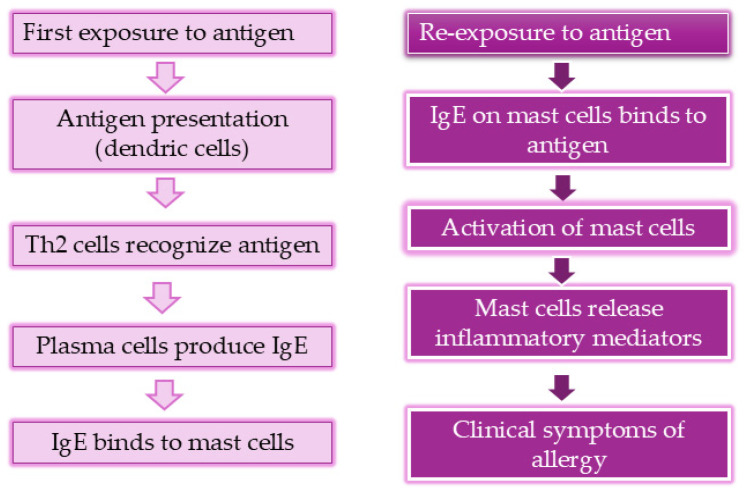
Mechanism of type I allergy reactions.

Type I hypersensitivity (Figure 1) reaction is characterized by an immune response to allergens and is known as IgE-mediated hypersensitivity. The first phase is the sensitization phase which begins with first exposure to the specific allergens, resulting in producing IgE antibodies. Upon re-exposure to the same allergens, inflammatory mediators are immediately released. The produced IgE antibodies occupy the high-activity receptors on the mast cells and basophil surface, and after the crosslinking between allergen and IgE antibodies, mast cells and basophils are activated resulting in the release of inflammatory mediators such as histamine, cytokines, and leukotrienes. The rapid release of these mediators causes clinical manifestations, from mild symptoms to life-threating reactions [12].

Antihistamines are the first line therapy for allergic diseases. They bind mainly to H1 receptors which are distributed in various cells. Though they are relatively successful in alleviating the symptoms of allergy, they can cause sedation [14]. The use of corticosteroids prevents and suppresses symptoms of allergic reactions. Despite good efficiency, their side effects, varying from oral candidiasis, dysphonia, nose bleeding, anosmia, to more severe systemic side effects, can be a barrier to adherence [15].

Leukotriene receptor antagonists are used for the prophylaxis and chronic treatment of asthma, and for the relief of allergic rhinitis symptoms. Common side effects include headache, abdominal pain, dyspepsia, and neuropsychiatric disorders [16,17]. Finally, bronchodilators are mostly used as rescue medicines in situations when despite regular therapy, symptoms persist [18,19]. 

Mast cells are key cells responsible for hypersensitivity allergic reactions (type 1) and they promote inflammation by releasing mediators such as histamine, proteases (pre-stored), cytokines, and eicosanoids (de novo synthesized). Another important regulator of pro-inflammatory cytokines expression is transcription factor nuclear factor kappa Β (NF-κΒ). Mitogen-activated protein kinases (MAPKs) and signal transducer and activator of transcription (STAT) are also important factors in immunoglobulin E (IgE)-dependent activation of mast cells [20,21].

Lymphocytes B and T, basophils, and eosinophils are also crucial for the development of allergic reaction. After the first exposure to the allergen, T cells (T helper type 2-TH2) are activated which causes the release of different cytokines, mainly interleukin (IL)-4 and IL-13. After the cytokines stimulate B lymphocytes, IgE antibodies are generated. The generated IgE is specific for the allergen [6,22].

Although allergies are generally not curable diseases, medicines can alleviate the symptoms of the disease. The treatments usually include antihistamines, steroids, antagonists of leukotriene receptor, bronchodilators, and finally immunotherapy.

Immunotherapy (preventive treatment with allergens) is the only form of treatment that leads to a significant reduction in symptoms; it can redirect the course of the disease, reduce the appearance of new sensitizations, and reduce future reactivity to a specific allergen [23]. Despite the numerous drugs used to control the symptoms of allergic diseases, many patients do not respond well to therapy due to side effects or lack of expected effects. Immunotherapy, as the best option for the treatment of allergic diseases, also does not find good cooperation among patients due to its long duration, potentially dangerous side effects, and high cost [24,25]. 

Considering all the above, herbal medicines are emerging as a potential source of drugs to relieve allergy symptoms without the unwanted side effects. The molecular mechanisms underlying allergic reactions are very complex, which open numerous possibilities for drug development. Herbal drugs, as a complex mixture of chemically diverse components, provide a wide range of possibilities for acting on individual allergic reactions pathways and are capable of multitargeting the immune system [26].

*A. membranaceus*, *N. sativa,* and *P. frutescens* have the potential to modulate immunological reactions to allergens, and therefore they are used as anti-allergic agents.

The ethnomedicinal use of these three species has been well documented. The root of *A. membranaceus* has been used in ethnomedicine of China, Greece, and Korea as remedy against body weakness and digestive system disorders, dyspepsia, hypertension, common cold, and for blood circulation [27]. Besides being used as adjunctive therapy for common colds and influenza, it is also known as preparation for enhancing the immune system and to stimulate the body’s endurance. Other traditional usages of *A. membranaceus* include chronic diarrhea, oedema, abnormal uterine bleeding, nephritis, chronic bronchitis, postpartum urine retention, and leprosy [28,29]. The ethnomedicinal uses of *N. sativa* seeds comprise its stomachic, laxative, carminative, and galactagogue activity. It was also used to alleviate headache, cough, and asthma to counter inflammation and fever, against ascites and jaundice, as well as to expel kidney stones [30]. The leaves, stems, and seeds of *P. frutescens* have been recorded in ethnomedicinal use. The leaves were mainly used for asthma, cough, colds, flu, chest stuffiness, vomiting, constipation, abdominal pain, and for promoting stomach function. The seed is used for arthritis and earache, while stems were known as analgesic and anti-abortion agents [31].

The aim of the work is to present the potential of herbal drugs with a traditional application to alleviate allergy symptoms, with special emphasis on *A. membranaceus*, *N. sativa*, and *P. frutescens*. Table 1 gives a short overview of botanical and common names of selected species. These species have been selected due to their availability on the market as dietary supplements/nutraceuticals. The review of the literature will include recent works that support the mechanisms of the anti-allergic effect of the mentioned plant species, as well as recent clinical studies.

## 2. Materials and Methods

A literature search was conducted for reports of preclinical and clinical studies. The databases employed for data collection are Scopus, PubMed, Web of Science, Springer, and Google Scholar. A comprehensive electronic search employed the following queries, individually or in combination: immunomodulation, allergic reaction, hypersensitivity, rhinitis, asthma, herbal drugs, *Astragalus membranaceus* Fisch. ex Bunge, *Nigella sativa* L., and *Perilla frutescens* (L.) Britton, in vivo studies, and clinical studies. Relevant articles were reviewed, and the most recent ones were preferably cited. The criterion for inclusion of in vivo preclinical and clinical studies in the manuscript was that they were preferably not older than 10 years.

## 3. Botanical Characteristics and Phytochemical Composition of *A. membranaceus*, *N. sativa,* and *P. frutescens*

*A. membranaceus* is distributed in China, Mongolia and Russian federation, South America, and Africa. It is a perennial herb, approximately 50–150 cm tall, with a straight, long, cylindrical root. Size of the root is 20–50 cm. Its stems are erect, with branches at the upper part, and are quilted with pubescence. Petiole base is odd-pinnate and alternate with lanceolate stipules, 25–37 leaflets, and broadly elliptical small leaves. The leaf apex is short-acuminate, base cuneate, entire, with both sides having long white pubescence [29,43]. The main reported phytochemicals in *A. membranaceus* are triterpenes, polysaccharides, flavonoids, and saponins. Main components are polysaccharides, formononetin (isoflavone), quercetin (flavone), and astragaloside IV (triterpene) [44].

*N. sativa* is distributed globally across the Middle Eastern Mediterranean region, Central and Southern Europe, Russian federation, Northern Africa, and Asia. *N. sativa* is an erect, herbaceous flowering plant with a stiff and multi-branched stem and a well-developed taproot [30]. It is an annual flowering plant, 20–90 cm tall, with finely divided leaves, the leaf segments narrowly linear to threadlike. The flowers are white, yellow, pink, pale blue or pale purple, delicate, with 5–10 petals. The fruit is a large and inflated capsule composed of 3–7 united follicles, each containing numerous small and black, rounded and streaked seeds [30]. Phytoconstituents from *N. sativa* are fixed oil with linoleic, oleic, myristic and palmitic acid, and volatile oil with thymoquinone, p-cymene, dithymoquinone (nigellone), carvacrol, and thymol. These volatiles are considered as main constituents responsible for biomedical activity of the plant. Other compounds found in *N. sativa* are saponins (α-hederin), polyphenols (kaempferol, quercetin, and rutin), as well as alkaloids (nigellimine and nigellidine) [45]. 

*P. frutescens* is widely distributed in East Asian countries, such as Japan, China, Korea, and Vietnam. It is a freely branching annual herb up to 150 cm tall. Stems are four-sided, covered with short hairs, and leaves are glossy and downy-haired, ovate, opposite, green to purple with toothed, crisped, laciniate, palmate, or serrate margins. Flowers are small, bell-shaped, white or purple and the seeds are small and globular [31,46].

**Figure 2:**
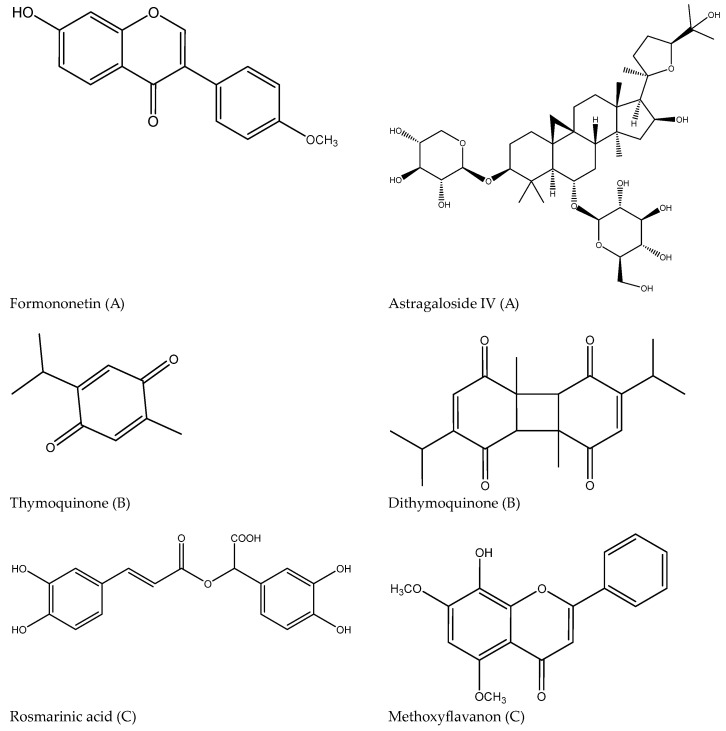
Major components with immunomodulatory activity isolated from *A. membranaceus* (**A**), *N. sativa* (**B**), and *P. frutescens* (**C**).

*P. frutescens* is a rich source of different types of phytoconstituents: terpenoids, flavonoids, phenolic acids, anthocyanins, coumarins, phenylpropanoids, sitosterols, and neolignans. However, mainly polyphenols were found to have anti-allergic activity—rosmarinic and caffeic acid, luteolin, and methoxyflavanon (8-hydroxy-5,7-dimethoxyflavanone) [46,47]. Figure 2 presents some constituents with immunomodulatory activity.

## 4. Anti-Allergic Activity of *Astragalus membranaceus*

*A. membranaceus* (Fabaceae) is a well-known traditionally used herbal drug with saponins and polysaccharides as main active compounds. Besides these compounds, *A. membranaceus* contains flavonoids, amino acids, and trace elements. So far, more than 100 compounds have been identified [48]. *A. membranaceus* has been proven to have various biological activities, namely, anti-inflammatory, anti-cancer, anti-diabetic, and antioxidant. Many studies have found that *A. membranaceus* has a therapeutic effect on allergic diseases, especially rhinitis and asthma [49]. The most used plant part is root, which is traditionally used as diuretic, anti-aging agent, and anti-hypertensive. However, more recent studies speak in favor of significant immunomodulatory effects. The large number of studies have focused on immunomodulatory activity of *A. membranaceus* polysaccharides and confirmed that it improves macrophage function and enhances phagocytosis of macrophages, promotes the activity of natural killer cells, dendritic cells, as well as T and B lymphocytes [50,51,52,53]. Astragalus polysaccharides are the most important natural active component of *A. membranaceus* and exert multiple pharmacological effects [52]. Owing to its low toxicity and side effects, it has been widely utilized [54]. Another valuable compound from *A. membranaceus* is astragaloside IV, a monomer isolated from total saponins, and an important component for evaluating the quality of *A. membranaceus*. Recent studies have revealed astragaloside IV as a potent compound for asthma treatment [55]. Table 2 summarizes recent studies that elucidate molecular mechanisms of action in the in vivo models on different allergic symptoms, as well as clinical studies. Many pre-clinical in vivo studies have been conducted to reveal mechanisms of action and effects on the allergy symptoms, both for *A. membranaceus* extracts and their components. The most used in vivo model was on ovalbumin (OVA)-sensitized mice. A study by Zhang et al. [56] estimated the potential of *A. membranaceus* polysaccharides (APSs) as an add-on therapy on an OVA-induced asthmatic mice model (25 µg intraperitoneally, inhaled 6% OVA solution). The mice were divided into four groups: control group (without OVA), budesonide group, budesonide + APS group, and untreated asthma group. The applicated dose of APS was 100 mg/kg body weight/day injected intraperitoneally. Results revealed that the mice treated with budesonide and APS showed alleviated airway resistance, dendritic cells were reduced, and NK cells as well as Treg cells were increased. They also had improvements in IL-4 and IL-10 mRNA and protein levels. These findings imply that APS as add-on therapy have the potential to relieve clinical symptoms of bronchial asthma. A similar study [57] confirmed these results, and improved lung function by inhibiting airway hyperresponsiveness, airway remodeling, and fibrosis. The *A. membranaceus* extract used in this study was standardized to 16% polysaccharides. Another compound from *A. membranaceus* astragaloside IV was also confirmed to have anti-asthmatic activity. Tested on OVA-sensitized mice (40 µg), in the doses of 10, 20, and 40 mg/kg, it reduced IL-4, IL-5, and IL-17 levels, increased INF-γ levels, and inhibited TORC1 activity [58].

APS were also prepared with chitosan microsphere to be suitable for nasal application. Rats were stimulated with OVA (0.3 mg/mL with aluminum hydroxide gel, 30 mg intraperitoneally; 50 mL 25 mg/mL once a day to the nasal cavity) to produce allergic rhinitis symptoms. Rats were administered with different doses of APS with chitosan (5, 10, 15 mg/kg) and compared to the control and budesonide groups. The APS with chitosan provided reduced allergic symptoms, eosinophils infiltration, and expression of IL-4, indicating excellent potential for the treatment of allergic rhinitis [59] Xu et al. [60] investigated the potential of APS on inflammatory pathways to treat symptoms of allergic rhinitis. Allergic rhinitis was induced by OVA (0.3 mg/30 mg aluminum hydroxide intraperitoneally; 50 µL 5% OVA intranasal administration), and the animals were divided into four groups: control, OVA, loratadine (0.9 mg/kg), and APS (400 mg/kg) group. APS alleviated the nasal symptoms and reduced the number of eosinophiles and cytokine levels (IL-4, IL-5, Il-6, IL-13, and TNF-α). This study drew attention to the impact of APS on two new inflammatory pathways—NLRP3 inflammasome and NOD2/NF-κΒ signaling pathway—responsible for the occurrence of allergic rhinitis. APS inhibited NLRP3 inflammasome activation and decreased NO2D expression and blocked the phosphorylation of NF-κΒ. These findings confirmed APS as potent anti-allergic agents. One of few studies investigating dry root extract of *A. membranaceus* was made on OVA-induced allergic rhinitis in mice. The animals were divided into four groups: control, OVA, *A. membranaceus* (25, 50, and 100 mg/kg/day), and dexamethasone (0.5 mg/kg/day) group. The mice were immunized by OVA (75 mg intraperitoneally, on day 0, 7, and 14; and with nasal application on OVA (200 µg/20 mL PBS) on day 21 and 42. The authors concluded that IgE and proinflammatory cytokines (IL-4, IL-6, and IL-13) have decreased in a dose-dependent manner. Levels of INF-γ and IL-10 were increased, similar to the proportion of CD4+CD25+Fox3+ T cells in the spleen and nasal lymphoid tissue. These kinds of T cells regulate autoimmune response, but they also have a role in regulating allergic diseases [49]. Recent study from He et al. [61] confirmed previous findings on APS activity in allergic rhinitis. In evaluating these studies, we must see several issues that prevent the comparison of studies. Firstly, there is a lack of analytical methods concerning *A. membranaceus* extract or APS quantification. Biologically active compounds in plant material can vary considerably resulting in different activities. More precise evaluation of tested plant material would contribute to the uniformity and thus the reliability of the obtained results. The second problem is the diversity in OVA regimen to induce allergic rhinitis/asthma. The last issue is the difference in used doses of *A. membranaceus*. However, despite all the obstacles, *A. membranaceus* has justified its traditional use as an anti-allergic compound, but more in vivo studies, preferably with the same protocol, are needed to confirm the exact molecular mechanisms and activity in allergic diseases.

Clinical studies confirmed the activity of *A. membranaceus* on allergic rhinitis and allergic asthma. However, we can still see the discrepancy between the dosage used in clinical studies. It is encouraging that both polysaccharides and astragaloside have the potential to ameliorate allergic symptoms (Table 2), and hopefully future studies will confirm and define the exact activity of different *A. membranaceus* products [62,63].

## 5. Anti-Allergic Activity of *Nigella sativa*

*N. sativa*, or the black cumin, originates from southwest Asia and belongs to the Ranunculaceae family. The most commonly used parts of the plant are seeds, more precisely, the oil obtained by pressing the seeds. The traditional use of *N. sativa* is known for many different illnesses, from airway diseases (asthma and cough), headache, infections (fever and influenza), and inflammatory diseases to hypertension and digestive tract disorders. Many ancient cultures used *N. sativa* oil for allergies. *N. sativa* seeds contain fat, proteins, carbohydrates, and high levels of carotene and minerals [64,65]. Today, the Pharmacopoeia of Inda recognizes *N. sativa* seed powder as a remedy for bowel and indigestion problems [45]. Although it is traditionally used mainly in the countries of the Middle East, *N. sativa* is increasingly researched and used in Western countries. The oil is a rich source of unsaturated fatty acids (linoleic and oleic). The saturated fatty acids, palmitic, and stearic are also present. Besides fatty acids, *N. sativa* seeds contain alkaloids, saponins, tocopherols, phytosterols, flavonoids, and essential oil which has the most health-promoting properties due to the most bioactive compound thymoquinone. Other valuable essential oil constituents are *trans*-anethole, *p*-cymene, limonene, α-thujene, dithymoquinone (nigellone), thymohydroquinone, carvacrol, and β-pinene [64,66,67,68]. The composition of essential oil may vary due to genetic composition, geographical distribution, methods of plant drying and essential oil isolation, climatic and seasonal composition, harvesting season, etc. The *N. sativa* essential oil is expected to be rich in thymoquinone, reaching values of 70–80%, but we can see that individually isolated oils contain only 0.79% [45,69]. The stated reasons make it much more difficult to standardize preparations, which results in large differences in preclinical and clinical trials and creates a false impression of the ineffectiveness of herbal preparations. 

Different fractions of *N. sativa* oil have been subjected to extensive antiradical and anti-inflammatory assays and showed potential to inhibit proinflammatory cytokines in a concentration-dependent manner [70].

Thymoquinone has been proven to have an influence on proinflammatory cytokines, inflammatory signaling pathways (NF-κΒ), signal transducer and activator of transcription 3 (STAT), mitogen-activated protein kinase (MAPK), peroxisome proliferator-activated receptor-γ (PPAR-γ), which speaks in favor of the effectiveness in treating allergy symptoms [71,72]. 

Günel et al. [73] have conducted an extensive in vivo study on the impact of thymoquinone on allergic rhinitis. The animals were divided into six groups: control group, OVA group, corticosteroid group (dexamethasone 1 mg/kg), healthy animal group treated with thymoquinone (10 mg/kg), and two animal groups with rhinitis treated with thymoquinone (3 and 10 mg/kg). The allergic rhinitis was induced by intraperitoneal injection of 0.3 mg OVA and 30 mg of aluminum hydroxide, and by intranasal dripping of 2% OVA, two or three drops per treatment. Specific IgE and IL-1β were significantly lowered by the dose of 10 mg/kg of thymoquinone, and both doses of thymoquinone (3 and 10 mg/kg) inhibited production of IL-4, IL-10, TNF-α, and decreased the eosinophil count in nasal mucosa. Similar study was performed on OVA-sensitized rhinitis in mice (0.3 mg OVA and 30 mg of aluminum hydroxide intraperitoneally; 30 µL 1 mg/mL OVA was instilled into both nostrils), where animals were treated with 2 mL/kg *N. sativa* oil or with mometasone furoate (5 µg/kg). Treated animals had lower nasal scratching frequency and no inflammation signs. Additionally, both treated groups had decreased eosinophil infiltration, cilia loss, chondrocyte hypertrophy, vascular proliferation, which confirmed that *N. sativa* oil was as effective as mometasone furoate [74]. However, the oil was not subjected to previous phytochemical characterization. An interesting animal model of asthmatic rats was performed by using smokeless tobacco exposure. The rats exposed to the smokeless tobacco showed enhanced lung inflammation accompanied by increased production of IL-4 and nitric oxide. *N. sativa* oil in doses of 4 mL/kg managed to decrease those proinflammatory parameters in OVA sensitization, smokeless tobacco administration, or both [75]. Numerous preclinical in vitro and in vivo studies of *N. sativa* paved the way for clinical studies. In the last decade, we can find clinical studies which evaluated the efficacy of *N. sativa* on allergic rhinitis and asthma. A clinical study investigating the topical application of *N. sativa* oil included 38 patients with allergic rhinitis, who were taking 2 drops of oil nasally, three times per day, during six weeks. The other 30 patients were in the control group. The patients who were taking *N. sativa* oil were divided into mild, moderate, and severe groups, according to the symptoms. Results of the study were very promising; all the patients from the mild group were free of symptoms, 68.7% patients from the moderate group, and 58.3% from the severe group were also symptom-free. Adverse effects were recorded only as nasal dryness [76]. *N. sativa* oil was also assessed as an add-on therapy in children with asthma. Regular therapy comprised inhalation of β2 agonist for intermittent asthma and β2 agonist + corticosteroid inhalation for persistent asthma. In the treatment group, children were given 15–30 mg/kg/day of oil for 8 weeks. There was no difference in asthma control test scores and Th1/Th2 balance between active and control groups, but the *N. sativa* oil group managed to decrease IL-4 and increase INF-γ levels [77]. Children with asthma were subjected to the evaluation of Th17/Treg balance after being supplemented with *N. sativa* oil as an add-on therapy, also in doses 15–30 mg/kg/day. The group with add-on therapy had a lower Th17/Treg ratio and better score on the asthma control test [78]. Another study evaluated *N. sativa* oil capsules (500 mg/twice per day) standardized to 0.7% of thymoquinone as an add-on therapy on routine asthma medications in adult asthmatic patients. Supplementation with *N. sativa* oil capsules enhanced the control of asthma symptoms and reduced blood eosinophilia [79]. Salem et al. [80] found that *N. sativa* whole ground seeds (1 or 2 g/day) along with maintenance-inhaled therapy improved pulmonary function and inflammation in partly controlled asthma. This was confirmed by increased FEF 25–75% (forced mid-expiratory flow) and FEV1 (forced expiratory volume), and decreased FeNO (fractional concentration of exhaled nitric oxide) and IgG. Table 3 summarizes in vivo and clinicals studies on *N. sativa*. Despite the high therapeutic potential, there is a lack on safety studies. A study by Thomas et al. [81] provided insight into the clinical safety of *N. sativa* oil formulation containing 5% of thymoquinone. Seventy healthy subjects were subjected to 200 mg/day of the above-mentioned formulation for a period of 90 days. After this period, extensive biochemical and hematological parameters were analyzed, and adverse effects were assessed. No signs of changes in biochemical parameters relevant to liver and renal function were identified, but significant reduction in total cholesterol, LDL, VLDL, and triglycerides was observed. However, the investigated formulation was proven to be safe for daily human consumption, and therefore can be evaluated for various pharmacological activities.

## 6. Anti-Allergic Activity of *Perilla frutescens*

*P. frutescens* is cultivated mostly in East Asia and belongs to the Lamiaceae family. The plant is rich in versatile phytochemicals, namely, flavonoids, alkaloids, phenylpropanoids, terpenoids, coumarins, which has been detected in *P. frutescens* root, seeds, and leaves. Due to the rich composition of phytochemicals, *P. frutescens* exhibits numerous pharmacological properties such as anti-inflammatory, neuroprotective, antioxidant, anti-cancer, and antidepressant activity, which makes it a good candidate for treatment of various diseases like diabetes, cardiovascular, and neurodegenerative diseases and allergic disorders. The traditional use of *P. frutescens* includes the preparation of tea from the leaves of the plant, as well as the use of essential oil from the leaves and oil extracted from the seeds [46]. *P. frutescens* leaves are rich in flavonoids (luteolin, apigenin, scutellarin, scutellarein), phenolic acids (rosmarinic, caffeic, chlorogenic, protocatechuic, gallic, ferulic acid), anthocyanins, and coumarins [82,83,84]. The bioactive compounds from *P. frutescens* have been proven to have an anti-allergic effect. Oh et al. [85] tested the ethanolic extract of *P. frutescens* along with rosmarinic acid on OVA-sensitized mice to evaluate the potential of alleviating symptoms of allergic rhinoconjunctivitis. *P. frutescens* extract and rosmarinic acid decreased the number of nasal, ear, and eye rubs, reduced IgE and histamine levels, and inhibited protein levels and mRNA expressions of IL-1β, IL-6, and TNF-α. Infiltration of mast cells and eosinophiles was also decreased. The above results clearly point to the potential of *P. frutescens* in alleviating the symptoms of allergic rhinoconjunctivitis. Table 4 summarizes selected in vivo and clinical studies. A more recent study has focused on the 8-hydroxy-5,7-dimethoxyflavanon, the component found in *P. frutescens* leaves, as a potential compound with anti-allergic activity. These assumptions were confirmed as 8-hydroxy-5,7-dimethoxyflavanon inhibited IgE-mediated histamine release in rat basophilic leukemia cells and suppressed allergic rhinitis symptoms in rats stimulated with Japanese cedar pollen allergens. The authors concluded that 8-hydroxy-5,7-dimethoxyflavanon directly acts on IgE–mast cell axis and negatively regulates hypersensitivity reactions but does not affect systemic immune response due to unaffected IgG1 and IgG2 antibody responses [47]. HPLC analysis of *P. frutescens* methanol extract revealed caffeic and rosmarinic acid, scutellarin, luteolin-glucoside, luteolin, quercetin, and apigenin as the main components. The tested extract showed a very good effect on the OVA-induced asthma model in mice, and the results of experimental and network pharmacology indicated that the anti-asthmatic mechanism of *P. frutescens* is through airway inflammatory and immune signal pathways and could be associated with the inhibition of extracellular signal-regulated kinases (ERKs), c-Jun N-terminal kinase (JNK), and p38 phosphorylation on the mitogen-activated protein kinase (MAPK) pathway [86]. Although the in vivo studies available in the last 10 years for the *P. frutescens* are quite scarce, they nevertheless speak in favor of its effective anti-allergic activity. It is encouraging that there are also clinical studies for the anti-allergic effect of this species. The effectiveness of a preparation containing *P. frutescens* extract in combination with quercetin and vitamin D (Lertal^®^) on allergic rhinoconjunctivitis was clinically tested. Two clinical studies were conducted on children in order to evaluate the potential of *P. frutescens* as an add-on therapy for allergic rhinitis. Through phases I and II of clinical trial, the activity of this nutraceutical has been confirmed by prevention of the worsening of clinical symptoms, and reduction in rescue drugs usage with no adverse effects [87,88]. In the study conducted in 2021, a dietary supplement was examined as a complementary therapy to standard therapy in patients with moderate to severe seasonal allergic rhinoconjunctivitis. Patients were divided into four groups: the first group received antihistamine nasal spray for 30 days, then *P. frutescens* supplement was added for the next 30 days; the second group received antihistamine nasal spray and corticosteroid nasal spray for 30 days, and again for the next 30 days the supplement was added to the therapy; the third and fourth groups were using antihistaminic and corticosteroid, respectively for the whole period of 60 days. The study aimed to evaluate the potential of *P. frutescens* supplement as an additional therapy in cases when the allergic reaction is very strong, and both intranasal antihistamine and corticosteroid are not sufficient to completely inhibit the allergic reaction. As expected, the results showed that within the first 30 days results were better in the group with patients who were treated with combined therapy (antihistamine and corticosteroid). In the next 30 days, the best results were recorded in the group with combined therapy and *P. frutescens* supplement, with significant reduction in all seasonal allergic rhinoconjunctivitis symptoms; the total symptom score was decreased by 37% [89]. The obtained results open the possibility of reducing standard topical therapy, which often causes side effects in patients (especially the use of corticosteroids). Morgana et al. [90] continued with evaluation of Lertal^®^ in adult patients with grass pollen-induced mild persistent asthma and rhinitis. All patients received corticosteroid and antihistamine and were divided into two groups: group A taking the *P. frutescens* supplement and group B using the predetermined therapy. Patients from group A had less severe bronchial and rhinitis symptoms, better asthma control with higher FEV1, and less usage of short-acting beta-2-agonists. Nasal eosinophils count was also decreased.

Although there are recent clinical studies that support the effectiveness of the *P. frutescens* on allergic rhinitis and asthma, supplementary clinical studies are needed in order to demonstrate the therapeutic effects and effective dosage. 

## 7. Toxicological Studies

*A. membranaceus*, *N. sativa,* and *P. frutescens* have been widely distributed on the market in the form of food and nutraceutical supplements. They are used in clinical practice for several years and comprehensive safety and toxicity studies are not yet conducted. So far, most of the available studies are conducted on animal models.

Scientists have focused on toxicity of secondary metabolites, and astragaloside was proven to be toxic above a dose of 1 mL/kg on an animal model. Acute toxicity, subacute or subchronic toxicity, genotoxicity, or immunotoxicity have not been observed. Astragalus was also evaluated on rats, who were treated with 0–150 mg/kg/day for a period of 91 days with no treatment-related deaths observed. Similar subchronic study was conducted on astragalus extract for 13 weeks with no deaths or toxic reactions [29]. Subchronic toxicity of astragalus extract was also observed on rats and beagle dogs to evaluate a safety dosage range in clinical application. The study demonstrated that the safety dosage range was 5.7–39.9 g/kg for rats, and 2.85–19.95 g/kg for dogs [43]. *N. sativa* oil showed no toxicity in doses up to 10 mL/kg in rat model. Hepatic toxicity of the *N. sativa* methanol extracts was tested at 6 g/kg/day for 14 days and revealed no abnormal activity of liver enzymes. The supplementation with 1 g/kg for 28 days produced no changes in liver enzymes or on liver function [30]. Another study investigated toxicity of thymoquinone and showed that lower doses (6 mg/kg/day) were well tolerated in animal model, but higher doses (8 mg/kg/day) resulted in death or signs of peritonitis. Thymoquinone was also proven to be genotoxic in high (80 mg/kg) doses [91]. *P. frutescens* has been associated with atypical interstitial pneumonia caused by perilla ketone from the seeds. The acute toxic dose of essential oil was 3 g herb/kg after intragastric administration on a mouse model [92]. Subchronic study was performed at doses of 3, 6, and 12 g/kg/day of Perilla seed oil for 90 days on dogs. In high and moderate doses, changes in hematology and serum biochemistry parameters, as well as histopathology of liver and lymph glands were observed. Dose of 3 g/kg/day was found to have no adverse effect after oral administration [93].

The disadvantage of the conducted studies is the non-uniformity of the studied doses and the duration of the studies. However, it is encouraging that no significant side effects were recorded in clinical studies of effectiveness, which indicates good tolerability of these species.

## 8. Conclusions

Nowadays, we are aware of the increase in allergic diseases that we associate with the modern way of life. Pollen allergies are leading among allergic diseases, and are manifested through allergic rhinitis, allergic conjunctivitis, and allergic asthma.

Although the symptoms are mostly not life-threatening, they significantly affect the patient’s quality of life. A specific problem is the adherence of patients with asthma, who very often do not take the therapy as prescribed, mainly due to frequent side effects. Due to the above reasons, it is necessary to research potential natural medicines with anti-allergic potential, which can reduce the need for regular pharmacological therapy. *A. membranaceus*, *N. sativa,* and *P. frutescens* have very well documented preclinical evidence suggesting their potential to alleviate allergy symptoms. Clinical studies also confirm their effect, although we observe the non-uniformity of the tested doses, which makes it difficult to compare them. Therefore, although they undeniably have a positive effect on allergic diseases, additional studies with uniform doses and standardized preparations are necessary to further confirm their effectiveness.

## Figures and Tables

**Table 1:** Binomial and vernacular plant names.

Binomial Plant Name	Synonyms [32,33,34]	Vernacular Names [35,36,37,38,39,40,41,42]	
		Language	Name
*Astragalus membranaceus* Fisch. ex Bunge	*Astragalus mongholicus* Bunge	English	Astragalus, Mongolian milkvetch, Membranous milk vetch
		German	Chinesischer tragant, Mogul tragant, Türkistan tragant
		French	Astragale, Astragale de Mongolie
		Italian	Astragalo
		Mongolian	Khunchir
		Chinese	Huáng huā huáng qí, Huáng qí, Běi qí
		Korean	Hwanggi
*Nigella sativa* L.	*Nigella cretica* Mill. *Nigella glandulifera* Freyn & Sint ex Freyn *Nigella indica* Roxb. ex Flem. *Nigella truncata* Viv.	English	Nigella, black caraway, black seed, black cumin, fennel flower, nutmeg flower, Roman coriander, black onion seed
		German	Schwarzkummel
		French	Nigelle, cheveux de Venus, poivrette
		Italian	Cumino nero, seme benedetto
		Arabic	Habba Al-Sauda, habba Al-Barakah
		Turkish	Cork
		Persian	Siyah danech
		Indian	Kalarija, kalonji
*Perilla frutescens* (L.) Britton.	*Melissa cretica* [Soland.] *Ocimum frutescens* L. *Perilla frutescens* var. *laviniata* W.Mill. & L.H.Bailey *Perilla ocymoides* L. *Perilla* ocymoides *f. discolor* Makino *Perilla ocymoides f. purpurea* Makino *Perilla ocymoides f. viridicrispa* Makino *Perilla ocymoides f. viridis* Makino *Perilla ocymoides* var. *japonica* Hassk. *Perilla ocymoides* var. *purpurascens* Hayata *Perilla urticifolia* Salisb.	English	Perilla mint, purple perilla Chinese basil, wild basil, blueweed Joseph’s coat, rattlesnake weed
		German	Sesamblatt, Schwarznessel
		French	Sésame sauvage
		Italian	Basilico della Cina, shiso
		Indian	Bhanjira
		Chinese	Ye sheng bai su, bai su, nan su, qing ye su, zǐsū
		Thai	Nga mon, nga khi mon
		Japanese	Shiso, egoma
		Korean	Deulkkae, kkaennip

**Table 2:** In vivo and clinical studies of *A. membranaceus* extracts and bioactive components.

Type of Herbal Drug	Experimental Model	Results	Ref.
Astragalus polysaccharides	OVA-induced asthmatic mice 100 mg/kg	Promoted therapeutic activity of budesonide Reduced the number of dendritic cells Reduced levels of IL-4 and IL-10 Increased Treg cells	[56]
*A. membranaceus* extract	OVA-induced asthmatic mice 100 mg/kg	Reduced plasma IgE and pulmonary Th2-related cytokines (IL-13 and IL-4) Reduced smooth muscle actin (SMA) in lungs	[57]
Astragaloside IV	OVA-induced asthmatic mice 10, 20, and 40 mg/kg	Reduced IL-4, IL-5, and IL-17 levels and increased INF-γ levels in the BALF Inhibited mTORC1 activity	[58]
Astragalus polysaccharides	OVA-induced allergic rhinitis mice 5–15 mg/kg, nasal application	Reduced eosinophil infiltration and IL-4 expression in nasal mucosa	[59]
Astragalus polysaccharides	OVA-induced allergic rhinitis mice 400 mg/kg	Reduced Th2-related cytokines of IL-4, IL-5, IL-6 and IL-13 levels in serum and nasal mucosa tissue Inhibition of NLRP3 inflammasome	[60]
*A. membranaceus* dry root	OVA-induced allergic rhinitis mice 25, 50, and 100 mg/kg	Reduced nasal sneezing and rubbing symptoms Decreased eosinophil infiltration in nasal mucosa Decreased IgE, IL-4, IL-5, and IL-13	[49].
Astragalus polysaccharides	OVA-stimulated and -sensitized guinea pigs to produce allergic rhinitis symptoms 25, 50, and 100 mg/kg	Reduced sneezing and rubbing times of AR guinea pigs Suppressed OVA-sIgE, OVA-sIgG1, TNF-α, and IL-6 levels Increased CD25+Foxp3+Treg cell Down regulation of NF-kB	[61]
*A. membranaceus* oral solution (astragaloside A as main compound)	80 children with allergic asthma 10 mL/day, 6 months	Increased IL-10 Decreased TGF-beta, IL-2, IFN-γ, IL-4, IL-6 Increased FEV1 percentage	[62]
*A. membranaceus* root extract (40% polysaccharides)	48 patients with seasonal allergic rhinitis 80 mg/day	Decreased intensity of rhinorrhea No change in the specific serum IgE, IgG, and nasal eosinophils	[63]

**Table 3:** In vivo and clinical studies of *N. sativa* extracts and bioactive components.

Type of Herbal Drug	Experimental Model	Results	Reference
Thymoquinone	OVA-induced allergic rhinitis rat 3 and 10 mg/kg	Reduced IL-4, IgE, TNF-α, and IL-1β Reduced eosinophil filtration and edema	[73]
*N. sativa* oil	OVA-induced allergic rhinitis rat 2 mL/kg	Lower frequency of nasal scratching No inflammation was observed	[74]
*N. sativa* oil	OVA-challenged and smokeless tobacco exposed rats 4 mL/kg	Reduced IL-4 and NO production	[75]
*N. sativa* oil	68 patients with allergic rhinitis Topical application 2 drops/3 times per day	Relief of allergy symptoms	[76]
*N. sativa* oil	28 children with asthma 15–30 mg/kg as additional therapy	Increased INF-γ Decreased IL-4 No difference in Th1/Th2 ratio	[77]
*N. sativa* oil with 0.7% thymoquinone	80 patients with asthma 500 mg/twice per day	Improvement in mean asthma control test Reduction in blood eosinophils Improved FEV1	[79]
*N. sativa* whole ground seeds	76 patients with partly controlled asthma 1 and 2 g/day	Increased FEF 25–75% and FEV1 Improved PEF variability; decreased FeNO and serum IgE Increased serum IFN-γ; improved ACT score	[80]
*N. sativa* oil	28 children with asthma 15–30 mg/kg as additional therapy	Decreased Th17; increased Treg percentages Lowered Th17/Treg ratio Improved ACT score	[78]

**Table 4:** In vivo and clinical studies of *P. frutescens* extracts and bioactive components.

Type of Herbal Drug	Experimental Model	Results	Reference
8-hydroxy-5,7-dimethoxyflavanon	Japanese Cedar pollen allergens sensitized mice 1.5 mg/day	Suppressed allergic rhinitis symptoms	[47]
*P. frutescens* extract	OVA-sensitized asthmatic mice 80, 160, and 320 mg/kg	Reduced asthma symptoms score in a dose- dependent manner Increased amount of air inhaled or exhaled during breathing Reduced levels of expiratory time, airway obstruction index, and frequency of breathing	[86]
*P. frutescens* extract *+* quercetin + vitamin D	146 children 80 mg + 150 mg + 5 µg as an add-on treatment for rhinitis	Prevention of worsening of clinical rhinitis symptoms	[87].
*P. frutescens* extract *+* quercetin + vitamin D	128 children 80 mg + 150 mg + 5 µg as an add-on treatment for rhinitis (4 weeks) and alone (4–12 weeks)	Reduced risk of rhinitis exacerbations Reduced rescue medication use	[88]
*P. frutescens* extract *+* quercetin + vitamin D	70 patients 80 mg + 150 mg + 5 µg in combination with standard rhinitis topical therapy	Increased effects of standard topical therapy Reduced nasal and ocular symptoms	[89]
*P. frutescens* extract *+* quercetin + vitamin D	90 patients 80 mg + 150 mg + 5 µg/twice per day in combination with antihistamine and corticosteroid	Reduction in bronchial symptoms Improved asthma control Increased FEV1 Reduced SABA and NC use Reduced nasal eosinophils count	[90]
